# Developmental Alcohol-Specific Parenting Profiles in Adolescence and their Relationships with Adolescents’ Alcohol Use

**DOI:** 10.1007/s10964-012-9772-9

**Published:** 2012-05-22

**Authors:** Ina M. Koning, Regina J. J. M. van den Eijnden, Jacqueline E. E. Verdurmen, Rutger C. M. E. Engels, Wilma A. M. Vollebergh

**Affiliations:** 1 Department of Interdisciplinary Social Science, Utrecht University, P.O. Box 80.140, 3508 TC Utrecht, The Netherlands; 2 Trimbos Institute, Netherlands Institute of Mental Health and Addiction, Utrecht, The Netherlands; 3 Behavioural Science Institute, Radboud University, Nijmegen, The Netherlands

**Keywords:** Alcohol-specific parenting, Adolescents, Parenting profiles, Alcohol use, Longitudinal, Communication

## Abstract

Previous studies on general parenting have demonstrated the relevance of strict parenting within a supportive social context for a variety of adolescent behaviors, such as alcohol use. Yet, alcohol-specific parenting practices are generally examined as separate predictors of adolescents’ drinking behavior. The present study examined different developmental profiles of alcohol-specific parenting (rule-setting, quality and frequency of communication about alcohol use) and how these patterns relate to the initiation and growth of adolescents’ drinking. A longitudinal sample of 883 adolescents (47 % female) including four measurements (between ages 12 and 16) was used. Latent class growth analysis revealed that five classes of parenting could be distinguished. Communication about alcohol appeared to be fairly stable over time in all parenting classes, whereas the level of rule-setting declined in all subgroups of parents as adolescents grow older. Strict rule-setting in combination with a high quality and frequency of communication was associated with the lowest amount of drinking; parents scoring low on all these behaviors show to be related to the highest amount of drinking. This study showed that alcohol-specific rule-setting is most effective when it coincides with a good quality and frequency of communication about alcohol use. This indicates that alcohol-specific parenting behaviors should be taken into account as an alcohol-specific parenting context, rather than single parenting practices. Therefore, parent-based alcohol interventions should not only encourage strict rule setting, the way parents communicate with their child about alcohol is also of major importance.

## Introduction

Most youngsters initiate alcohol drinking during adolescence, going from irregular drinking patterns in early adolescence into more habitual patterns during middle and late adolescence (Poelen et al. 2005). The age at which adolescents start using alcohol is associated with several risks (e.g., alcohol abuse, brain damage, school performance; Behrendt et al. 2009; Brown and Tapert 2004). A vast amount of international studies consistently shows the importance of alcohol-specific parenting from early (Habib et al. 2010; Koning et al. 2010a) through middle (Van der Vorst et al. 2006) into late adolescence (Abar and Turrisi 2008). This has led to an increased interest in how parents can be targeted effectively in alcohol intervention programs. Parent-based interventions addressing restrictive parenting (e.g., monitoring, attitudes, rule-setting) with respect to alcohol use appears to be an effective method identified in several international studies (e.g., Koning et al. 2010b; Koutakis et al. 2008; Turrisi et al. 2009), whereas targeting parent–child communication reveal less promising findings (Turrisi et al. 2001, 2009; Wood et al. 2010). Yet, rules about alcohol should somehow be made explicit to the child via communication (Ennett et al. 2001). The relationship between quality and frequency of communication about alcohol and adolescents’ alcohol use are fairly inconsistent. Though a higher quality of communication generally relates to lower rates of drinking, the relationship of frequency of communication with alcohol use varies from positive to negative. It is likely that the influence of frequency of communication on adolescents’ drinking depends on the context wherein these conversations take place. Therefore, in this study we examine how rules and quality and frequency of communication about alcohol use coincide and relate to alcohol use in adolescents. Refined knowledge about the way rules about alcohol should be conveyed could enhance parental interventions.

As stated previously, different alcohol-specific parenting practices are likely to coincide. That is, a parenting practice (e.g., setting rules) is not likely to act on its own, but is related to other (alcohol-related) parenting practices. Yet, only a few studies report on the relations between parenting practices. For example, communicating more frequently about alcohol is related to less restrictive rule-setting (Van den Eijnden et al. 2011), with stronger relationships in adolescents with higher drinking rates (Van der Vorst et al. 2010). However, Mares et al. (2011) showed a positive relationship between strict parenting and the frequency of communication; having a father with strict attitudes about alcohol was related to having a mother who communicated more often about alcohol. In addition, a higher quality of communication is related to a higher frequency, yet no relationship between quality of communication and rule-setting is found (Van den Eijnden et al. 2011). Van Zundert et al. (2006) did show, however, that more strict rule setting was related to higher levels of maternal emotional support, which can be seen as a proxy for the quality of communication about alcohol. Abar et al. (2011) found in their cross-sectional study that parents who communicate frequently about alcohol use and its consequences tend to engage more in parental monitoring and reported to have a better parent–teen relationship compared to parents who talked about alcohol use but did not discuss its consequences. Though current knowledge on the relationships between alcohol-specific parenting practices is inconsistent, overall the studies indicate that strict rules about alcohol are likely to coincide with a frequent and qualitative way of communication.

A large amount of data is available on the combination of general parenting practices, whether or not in relationship to alcohol use (Adalbjarnardottir and Haffsteinson 2001; Coley et al. 2008; Latendresse et al. 2009). For example, adolescents with a qualitative relationship with their parents, and whose parents are fairly strict, are less likely to engage in high risk drinking (Mallett et al. 2011). In general, parenting behavior that incorporates elements of authoritative parenting (Baumrind 1968), such as “reciprocity of communication” and “explanation of reasoning” (Darling and Steinberg 1993, p. 492), appeared to be most beneficial for a variety of adolescent behaviors, including alcohol use (Adalbjarnardottir and Haffsteinson 2001; Latendresse et al. 2009). Yet, it is important to examine parenting behavior specifically with respect to alcohol use since general parenting practices are found to be related less strongly to drinking behavior in adolescents than alcohol-specific parenting practices (Van Zundert et al. 2006). Moreover, Van Zundert et al. (2006) revealed that alcohol-specific rules intervene in the relationship between general parental control and adolescents’ drinking. Therefore, targeting alcohol-specific parenting is likely to induce more change in the actual drinking behavior in adolescents. More insight into the joint development of rules and communication about alcohol, therefore likely contributes to the refinement of interventions to foster healthier drinking behavior in adolescents.

## Current Study

This article addresses two research questions. First, can specific developmental parenting profiles based on rule-setting behavior and communication about alcohol be distinguished from early to mid-adolescence? And second, how do these parenting profiles relate to adolescents’ drinking? Based on previous research, it is hypothesized that restrictive rule-setting is likely to coincide with a higher quality and frequency of communication about alcohol (Abar et al. 2011; Mares et al. 2011; Van Zundert et al. 2006). Furthermore, it is expected that a combination of restrictive rules, high quality and frequency of communication is associated with lower levels of adolescent drinking. A longitudinal sample of 883 Dutch adolescents, including data from four different time points (between ages 12 and 15) is used. This enables the examination of developmental alcohol-specific parenting profiles and its relation to adolescents’ drinking over time.

## Method

### Design and Procedure

The current study is part of a larger alcohol prevention randomized trial conducted in the Netherlands (see Koning et al. 2009a, 2011) in which 19 schools were randomly selected and assigned to either of the three intervention conditions or to the control condition. For purposes of this study, only adolescents and parents who were assigned to the control condition were included in current analyses. In this way, the data are not affected by the interventions. Baseline data (T1) were collected at the beginning of the first high school year (September/October 2006). The first follow-up (T2) was 10 months later in June/July 2007, then again in June/July 2008 (T3) and June/July 2009 (T4). Trained research assistants administered digital questionnaires to adolescents in the classroom. Questionnaires for parents and a letter of consent were sent to their home addresses. This letter informed parents about the participation of the school in the project and parents were given the opportunity to refuse participation of their child (0.01 % refusal). Non-responding parents were reminded after 3 weeks by mail and after another 2 weeks by phone.

### Participants

Nine schools including 935 adolescents were selected to participate in the study. Due to initial non-response among adolescents (*n* = 29) and unreliable data on the alcohol measure (i.e., extreme responses; *n* = 23), 883 adolescents were eligible for analyses.

Table 1 depicts the characteristics of adolescents at baseline. The adolescent sample had a mean age of 12.19 (SD = 0.5) at baseline, including 53 % boys and 47 % girls, 60 % in lower secondary vocational education (lower education) and 40 % in higher general secondary and pre-university education (higher education). Almost one fifth of the adolescents (18 %) reported to live in a single-parent family, which is in accordance with the national percentage of 19 %. (CBS 2011). Adolescents drank an average of 0.69 alcohol drinks per week (Table 2).

**Table 1:** Characteristics of adolescents at baseline

Variable	
Male, *n* (%)	476 (52.5)
Age, years: mean (s.d.)	12.6 (0.46)
Low level of education, *n* (%)	360 (39.7)
Single-parent family, *n* (%)	159 (18)

**Table 2:** Average number of alcoholic drinks per week (SE) at waves 1–4

Wave	Alcoholic drinks (*M*, SE)
1	0.7 (3.6)
2	1.9 (8.4)
3	3.4 (12.3)
4	6.0 (13.1)

### Attrition Analyses

A total of 843 adolescents (95.5 %) at T2, 783 adolescents (88.7 %) at T3 and 764 adolescents (86.5 %) at T4 stayed in the program and completed the follow-up assessments after ten, 22 and 34 months respectively. A total of 618 parents at T2 (87.9 %), 532 parents at T3 (75.7 %) and 496 parents (66.7 %) at T4 participated in the study.

Attrition analyses on demographic variables and alcohol use indicated that responding adolescents at T3 and T4 were more likely to be younger (T3: *t* = 2.65, *p* = 0.01; T4: *t* = 2.73, *p* = 0.01), tended to follow lower education programs (T3: χ^2^ (1) = 18.24, *p* < 0.00; T4: χ^2^ (1) = 16.67, *p* < 0.001), and drank a lower average number of alcoholic beverages per week at baseline (T3: *t* = 4.67, *p* < 0.00; T4: *t* = 4.30, *p* < 0.00). At T2, no significant differences were found on these characteristics. At T2, adolescents of participating parents reported a significantly higher quality of communication (*t* = 3.79, *p* = 0.02). No other significant differences were found for rules and communication about alcohol.

### Measures

#### Adolescents’ Alcohol Use

Drinking behavior was measured by using the Quantity-Frequency measure (at T1 to T4). The Quantity-Frequency measure represented the average weekly alcohol use. Frequency was measured by asking the number of days the adolescent usually drank on weekdays (Monday to Thursday) and weekend days (Friday to Sunday) (Engels and Knibbe 2000). Quantity was measured by asking how many glasses of alcohol the adolescent usually drinks on a weekday and weekend day (Engels et al. 1999). Quantity-Frequency was computed by calculating the products of the number of days and the number of glasses, then summing the two products for weekdays and weekend days.

#### Rules About Alcohol

The degree of parental rule-setting regarding the adolescent’s alcohol use (at T1 to T4) was measured with a ten-item scale developed by Van der Vorst et al. (2005). Items included “I am allowed to have one glass of alcohol when one of my parents is at home”, “I am allowed to drink several glasses of alcohol when one of my parents isn’t home” and “I am allowed to drink alcohol at a party with my friends”. The mean of ten items rated on a 5-point scale from 1 (*never*) to 5 (*always*) reversely scored was used, i.e., higher scores indicating more rule-setting behavior. Cronbach’s alpha ranged from .81 to .94

#### Frequency of Communication About Alcohol

The frequency of communication about alcohol referred to how often in the past 12 months the parent had talked with the adolescent about specific alcohol-related issues (T1 to T4), such as the negative consequences of use, rules about alcohol use, discipline, telling the adolescent not to use, media portrayal of alcohol, and ways to resist peer pressure (Ennett et al. 2001; and translated and adapted by Van der Vorst et al. 2005). We reduced the scale to six items (cf. Spijkerman et al. 2008), including a 5-point scale from 1 (*never*) to 5 (*very often*). Higher scores indicate higher frequency of communication. Cronbach’s alpha ranged from 0.88 to .90.

#### Quality of Communication About Alcohol

The quality of communication about alcohol was measured at T1 to T4 by asking about the adolescents’ perceptions of the quality of communication about alcohol with their parents. The scale was developed for smoking by Harakeh et al. (2005) and was adapted for drinking (Spijkerman et al. 2008). Items included “My parents and I are interested in each other’s opinion regarding alcohol use”, “If my parents and I talk about alcohol, I feel understood”. The mean of six items rated on a 5-point scale ranging from 1 (*not at all*) to 5 (*very much*) was used. Higher scores indicate a higher quality of communication. Cronbach’s alpha ranged from 0.79 to .86.

All parenting measures were reported by the adolescent, as previous studies showed stronger and more consistent relations of adolescent-reported parenting behaviors than parent-reported parenting behaviors (Koning et al. 2010a).

### Strategy for Analyses

To analyze our first research question of whether different alcohol-specific parenting profiles can be distinguished, different classes were identified by applying Latent Class Growth Analysis (LCGA) in Mplus 5.0 (Muthén and Muthén 2007) to the list of three parenting behaviors (rules about alcohol, quality and frequency of communication about alcohol) measured at T1 to T4. LCGA is a person-centered statistical approach of identifying latent subgroups within a heterogeneous population that follow distinct trajectories over time for a given outcome that is measured repeatedly. The number of classes (i.e., naturally occurring subgroups) is estimated by modeling a range of class numbers and determining the best fit for the data set. Based on the assumption that the subgroups are homogenous, LCGA does not estimate the variability around each subgroup’s trajectory (Jung and Wickrama 2008); the variance of the intercepts and slopes are held at zero for simplicity in modeling. The goal of LCGA is to identify the smallest number of latent classes that adequately describes the associations among the observed variables. We started with the most parsimonious 1-class model and fitted successive models with increasing numbers of classes. Goodness-of-fit statistics were used to select the optimal model (Brown 2006). We compared successive models using the Sample Size Adjusted Bayesian Information Criterion (SSA-BIC), the Entrophy and the Vuong Mendell statistics. In addition, theoretical meaningfulness of classes in the various solutions was considered.

Next, a linear growth model (LGM) was estimated (Mplus 5.0; Muthén and Muthén 2007) based on the adolescent’s alcohol use reported at four time points over a four-year period (T1, T2, T3, T4). The alcohol use scores were negatively skewed; therefore, LGM was applied using a Poisson distribution with the adolescent’s alcohol use as count variables (Muthén and Muthén 2007). Different types of latent growth models were estimated to determine which model fit the data best (linear growth or quadratic growth). We used multigroup LGMs, with the parenting style classes as groups.

Last, descriptive data on the demographic variables were used to characterize the different parenting profiles. Missing data are handled in Mplus with a robust maximum likelihood estimator, which takes advantage of all available data rather than deleting cases with partially missing data in a listwise manner.

## Results

### Parenting Profiles

**Fig. 1:**
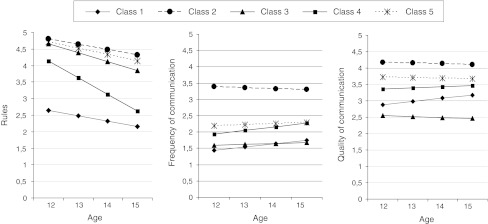
Developmental trajectories (intercept and slope) for rules and frequency and quality of communication about alcohol use for five classes of alcohol-specific parenting

Table 3 shows results for each of the LCGA model fit statistics. A five-class solution was identified to best fit the data, according to the SSA-BIC and the nearly significant Vuong-Lo-Mendell-Rubin likelihood ratio test (Nylund et al. 2007). The average class probabilities were high (.86–.96), which indicated that the participants were classified properly in their latent class. The intercepts and slopes of the latent variables comprising the five parenting profiles designed by LCGA are presented in Table 4 and graphically illustrated in Fig. 1.

**Table 3:** Criteria for deciding the number of classes

No. of classes	*H*	SSA-BIC	LMR LRT statistic	LMR LRT *p*-value
2	.74	23,183	1,150	.000
3	.81	22,664	632	.00
4	.80	22,223	356	.03
5	.80	22,004	239	.06
6	.83	22,188	131	.255

*SSA-BIC* Sample size adjusted bayesian information criterion, *H* entropy measure, *LMR LRT* Lo Mendell Rubin Likelihood Ratio Test

**Table 4:** Means and standard errors of intercepts and slopes of latent indicators (rules, frequency and quality of communication) for five parenting profiles

*N* = 883	Rules about alcohol		Frequency of communication		Quality of communication	
	Intercept	Slope	Intercept	Slope	Intercept	Slope
1. Permissive *N* = 25, 3 %	2.69 (.25)	−0.16 (.17)^†^	1.44 (.10)	0.10 (.07)^†^	2.88 (.19)	0.10 (.08)^†^
2. Authoritative *N* = 143, 16 %	4.80 (.03)	−0.16 (.02)	3.39 (.11)	−0.03 (.05)^†^	4.17 (.06)	−0.02 (.03)^†^
3. Authoritarian *N* = 160, 18 %	4.66 (.05)	−0.27 (.03)	1.59 (.09)	0.03 (.04)^†^	2.55 (.14)	−0.03 (.06)^†^
4. Decliners *N* = 161, 18 %	4.13 (.09)	−0.50 (.05)	1.94 (.07)	0.11 (.03)	3.37 (.09)	0.03 (.03)^†^
5. Moderately authoritative *N* = 393, 45 %	4.71 (.03)	−0.19 (.02)	2.20 (.06)	0.03 (.02)^†^	3.73 (.05)	−0.02 (.02)^†^

^† ^No significant change

Class 1 (3 %) was characterized by a low level of rule-setting at age 12 and revealed no significant change over time, indicating lenient parenting across adolescence. This same development was found for the frequency and quality of communication; fairly low scores at age 12 with no significant change over time. Class 1, therefore, could be termed *Permissive.* Class 2 (16 %) was characterized by a high level of rule-setting that showed a significant, yet minor decrease in strictness over time. Adolescents in this class reported high levels of frequency and quality of communication that remained stable over time. Class 2, therefore, was termed *Authoritative*. Class 3 (18 %) was characterized by a moderate level of strict rule-setting at age 12, with a moderately strong decrease in strictness over time. Furthermore, adolescents in this class reported a constant low level of frequency and quality of communication over time. Class 3 was defined as *Authoritarian*. Class 4 (18 %) was characterized by a moderately low level of strict rule-setting at age 12 and a strong significant decline over time. The frequency of communication was moderately high at age 12 and this significantly increased by age. A constant moderate quality of communication was reported. This class was referred to as *Decliners.* Class 5 (45 %) was characterized by a relatively high level of strict rule-setting at age 12, which significantly, yet slightly, declined by age. Frequency and quality of communication was moderately high and did not change over time. Descriptive data of the parenting profiles (see Table 5) revealed that classes 2 (*Authoritative*) and 5 (*Moderately Authoritative*) showed the lowest (respectively 46 and 48 %) and class 3 the highest (Authoritarian: 64 %) percentage of boys.

**Table 5:** Descriptive statistics of adolescents included in the five parenting profiles

	1.	2.	3.	4.	5.
Gender (% boys)	58.3	45.5	64.2	53.4	47.5
Educational level (% low education)	87.5	51.0	69.8	68.1	53.0

Descriptive data of the parenting profiles revealed that classes 2 (*Authoritative*) and 5 (*Moderately Authoritative*) showed the lowest (respectively 46 and 48 %) and class 3 the highest (*Authoritarian*: 64 %) percentage of boys. Furthermore, class 1 (*Permissive* parents) has the highest percentage adolescents that were in lower levels of education (88 %) followed by classes 3 (*Authoritarian*: 70 %), 4 (*Decliners*: 68 %) and 2 (*Authoritative*: 51 %) and 5 (*Moderately Authoritative*: 53 %).

### Alcohol Use Across Parenting Profiles

**Fig. 2:**
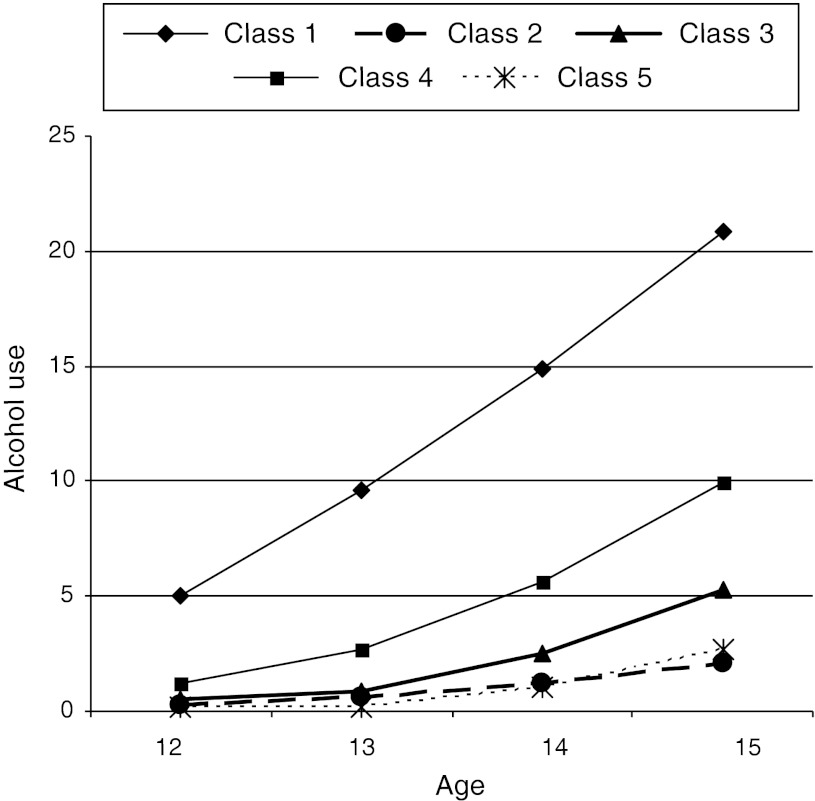
Development of alcohol use at wave 1 to 4 for each of the parenting classes

A quadratic growth curve model fitted the model best, showing the lowest BIC value. Table 6 shows the means and standard errors of intercept, slope and quadratic growth of adolescents’ alcohol use across the five parenting profiles. Figure 2 depicts graphical representations of the corresponding development of alcohol use for the parenting profiles.

**Table 6:** Means and standard errors of intercepts, slopes and quadratic slopes of adolescents’ alcohol use (weekly drinking) for five parenting profiles

Class (*N* = 883)	Intercept		Slope		Quadratic slope	
	*M*	SE	*M*	SE	*M*	SE
1. Permissive	5.04**	1.14	4.20	2.95	0.36	1.22
2. Authoritative	0.24*	0.10	0.19	0.28	0.14	0.13
3. Authoritarian	0.50**	0.14	−0.20	0.35	0.60**	0.17
4. Decliners	1.21**	0.25	0.81	0.60	0.71**	0.24
5. Moderately authoritative	0.19**	0.05	−0.42**	0.12	0.42**	0.06

* *p* < .05; ** *p* < .01

Class 1 (*Permissive* parenting) stands out based on the highest rate of drinking at age 12 (intercept) and the steepest increase over time. Class 4 (*Decliners*) includes adolescents with the second highest rate of drinking at first and over time, followed by class 3 (*Authoritarian*). All other classes have a similar level of drinking at wave 1, yet class 3 increases more steeply compared to classes 2 (*Authoritative*) and 5 (*Moderately Authoritative*).

## Discussion

Parents exert a consistent and strong influence on their child’s alcohol use throughout adolescence, in particular by setting alcohol-specific rules (Habib et al. 2010; Van der Vorst et al. 2006; Yu 2003). As a result, alcohol-specific rules are often a target in parent-based alcohol interventions, with favorable effects (e.g., Koning et al. 2010b; Koutakis et al. 2008; Turrisi et al. 2009). In line with knowledge on general parenting, research indicates the importance of setting rules in a supportive environment by, for example, having qualitative positive parent–child communication about alcohol (Mallett et al. 2011). Yet, studies show that targeting parent–child communication in alcohol intervention is not an effective way to change adolescents’ drinking behavior (Turrisi et al. 2001, 2009; Wood et al. 2010). Nevertheless, rules about alcohol should somehow be made explicit to the child via communication (Ennett et al. 2001). Currently, it is unknown how rules about alcohol coincide with the way parents talk about alcohol with their child and, in addition, what combination of rules and communication is most beneficial regarding adolescents’ drinking. More insight into the joint development of rules and communication about alcohol, contributes to the refinement of interventions to foster healthier drinking behavior in adolescents.

The first research aim in this study was to examine whether different developmental profiles of alcohol-specific parenting (rule-setting, quality and frequency of communication about alcohol use) could be distinguished. Results revealed the distinction of five alcohol-specific parenting profiles based on the level of rule-setting and quality and frequency of communication about alcohol reported at age 12–16. Inspection of these parenting profiles points at two findings that are worth further consideration. First, considering the development of the parenting behaviors across adolescence, results indicate an overall decline in strict rule setting across parenting profiles (except for the permissive parents), whereas how and how frequent communication about alcohol takes place appears to be fairly stable over time. The decline in strictness of parents during adolescence is in line with previous research on alcohol-specific (Monshouwer et al. 2008; Van der Vorst et al. 2006) and general parenting (Keijsers et al. 2009). Most likely, parents tend to become less strict with age, due to adolescents’ drive to gain (Masche 2010) and parents’ willingness to grant autonomy (Darling et al. 2006). Overall, adolescents reported no change in the communication they had with their parents about alcohol as they become older. Only those adolescents whose parents considerably became more lenient (*Decliners*), reported an increase in the frequency of communication about alcohol. It is speculated that in an attempt to lower their child’s alcohol use, a more frequent communication is a response of parents to the increasing level of alcohol use in their child as he/she becomes older (Van der Vorst et al. 2010). Contrary to previous alcohol research (e.g., Keijsers et al. 2009; Van der Vorst et al. 2010), our study demonstrates the stability of communication about alcohol during adolescence. Second, strict alcohol-specific rules tend to coincide with a high quality and frequency about communication. De Goede et al. (2009) examined developmental changes in adolescents’ perceptions of parent–child relationships and demonstrated that adolescents who perceive their parents as powerful are viewed as more supportive. This indicates that strict parenting can be considered as a form of parental involvement, which is reflected by the concurrent experience of strict parental rules with qualitative and frequent parent–child communication (cf. correlations found in previous studies; Abar et al. 2011; Mares et al. 2011; Van Zundert et al. 2006). It seems that a more frequent communication will do no harm when it occurs in a supportive context (high quality of communication and strict parents). This is exemplified by the fact that the quality and frequency of communication tend to go together: the better the quality of alcohol-related conversations, the more frequent these conversations are held. As hypothesized, the distinct alcohol-specific parenting profiles that were found in the current study confirm that a high level of strict alcohol-specific rule setting coincides with a quality and frequency of communication about alcohol, indicating that alcohol-specific parenting behaviors should be taken into account as an alcohol-specific parenting context, rather than single parenting practices.

The second aim of the study was to examine how these parenting profiles relate to the initiation and growth of adolescents’ drinking. Adolescents with parents who remained relatively strict and who had frequent and qualitative communication about alcohol during adolescence *(moderately authoritative parenting profiles*) were less likely get involved in drinking at age 12 and increase rapidly to higher levels of drinking (Adalbjarnardottir and Haffsteinson 2001; Latendresse et al. 2009; Mallett et al. 2011). Adolescents reporting low levels of strict rule-setting and communication about alcohol (*Permissive parenting)* were most likely to drink alcohol at age 12 and to accelerate quickly to higher levels of drinking with age. The combination of relatively strict parenting over time with low levels of communication (a*uthoritarian parenting profile)* points at the fact that strict parental rules are the most important parenting practice (Habib et al. 2010; Van der Vorst et al. 2006; Yu 2003). That is, the level of drinking initially corresponds to that of adolescents in the (moderately) authoritative parenting profiles, yet due to the unsupportive context wherein these rules are set, adolescents in the authoritarian profile increase their drinking more quickly over time than adolescents with (moderate) authoritative parents. The importance of strict rules about alcohol is also exemplified by the fact that a *declining parenting profile*, i.e., a strong decline in strict rule setting over time and a moderate level of communication, is distinguished. Adolescents with parents who have a declining parenting profile end up having the second highest level of drinking across adolescence. Thus, in regard to adolescents’ drinking at age 12–16, setting restrictive rules during adolescence is most effective when these rules are combined with high quality and frequency of communication.

To a large extent our findings are in line with the typology of general parenting styles defined by Baumrind (1968), who demonstrated that parenting style is characterized by level of control and support. In this study, we demonstrated the importance of these two dimensions in terms of alcohol-specific parenting. For example, alcohol-specific rules about alcohol and quality of communication about alcohol reflect, respectively, general control and support dimensions. However, little is known concerning how communication about alcohol can be viewed qualitatively (i.e., how can rules be conveyed in a qualitative way). In general—and supported by our results—it is likely that alcohol-specific rules should be clear and firmly enforced; more importantly, the reasoning behind the rules should be explained. At the same time, parents should express interest in their children’s needs and allow their children to question the rules (Stice et al. 1993). Further, more research is needed to gain a better understanding of how a high quality communication about alcohol can be achieved. In sum, though it is known that restrictive alcohol-specific parenting is a strong predictor of alcohol use in adolescents, this study is the first that demonstrated the relevance of setting strict rules in combination with qualitative and frequent communication about alcohol.

### Limitations

There are several limitations to address. First, adolescents’ alcohol use was based on self-reported data, whereas other methods such as cross-reports or diary reports may have yielded more reliable data. However, self-reports have been found to be fairly reliable (Koning et al. 2009b; Wagenaar et al. 1993), and other methods are rather expensive when using large samples. Second, the prevalence of *permissive* parents is fairly small. Yet, this group involves parents who show, from their offspring’s point of view, the most problematic behavior. In line with nearly all forms of extreme behavior, so also with respect to alcohol use in adolescents, a low prevalence is expected. Moreover, from a clinical perspective this group is of most interest. Third, in this article the uni-directional relationship between alcohol-specific parenting and adolescents’ drinking is assumed. Although studies demonstrate that alcohol-specific parenting predicts more strongly adolescents’ drinking, the effect of adolescents drinking on parenting also has been shown (e.g., Van der Vorst et al. 2006). Fourth, alcohol-specific parenting practices were reported by the adolescents. We should consider that reports of these practices might be related to child-specific characteristics (Tein et al. 1994), such as emotionality and/or personality, which in turn may also be related to the differences in alcohol use behaviors among the adolescents. Yet, it is the perception of parenting practices which seems to determine adolescents’ subsequent behavior. Fourth, as accounts for general parenting styles (Steinberg et al. 1991), the influence of alcohol-specific parenting practices may also be subject to contextual influences—that is, across cultures parents may have different goals for socializing their children and drinking alcohol at an early age may have a different meaning. This contextual limitation has implications for the generalizability of our findings. We should also take into account that parental rule-setting concerning alcohol use is considered to be more legitimate and thus accepted by adolescents than rules regarding personal matters such as clothing (Smetana 2000). The most effective parenting profile with respect to the use of alcohol may therefore differ for other risk behaviors.

### Conclusions

Current findings have several implications for practice as well as scientific understanding of adolescence. More insight has been gained with respect to the combination of alcohol-specific parenting behaviors and adolescents’ alcohol use. The relevance of restrictive rule-setting in combination with regular and qualitatively good communication about alcohol use is established. Practitioners working with parents in alcohol prevention programs therefore should focus not only on the relevance of the rule-setting but also on the importance that these rules will be conveyed regularly in an open communication style. In addition, both adolescents who end up drinking the highest amounts of alcohol and their parents should be targeted by alcohol prevention programs. A Dutch alcohol prevention program (PAS) succeeded at postponing the onset of drinking in adolescents (Koning et al. 2009a, 2011) by, amongst other things, increasing parents’ restrictive rule-setting (Koning et al. 2010b). The current findings underline the relevance of targeting alcohol-specific parenting behaviors. More information about the relevance of an open and regular style of communication in combination with restrictive rule-setting should be provided in this and other prevention programs. In line with knowledge on general parenting, the current study revealed the existence of an alcohol-specific parenting context wherein parents guide their children towards responsible drinking by setting strict alcohol-specific rules and having supportive parent–child communication about alcohol.
